# Genome-Wide MicroRNA Expression Analysis of Clear Cell Renal Cell Carcinoma by Next Generation Deep Sequencing

**DOI:** 10.1371/journal.pone.0038298

**Published:** 2012-06-20

**Authors:** Susanne Osanto, Yongjun Qin, Henk P. Buermans, Johannes Berkers, Evelyne Lerut, Jelle J. Goeman, Hendrik van Poppel

**Affiliations:** 1 Department of Clinical Oncology, Leiden University Medical Center, Leiden, the Netherlands; 2 Department of Human Genetics, Leiden University Medical Center, Leiden, the Netherlands; 3 Department of Medical Statistics, Leiden University Medical Center, Leiden, the Netherlands; 4 Department of Urology, Catholic University of Leuven, Leuven, Belgium; 5 Department of Pathology, Catholic University of Leuven, Leuven, Belgium; Baylor College of Medicine, United States of America

## Abstract

MicroRNAs (miRNAs), non-coding RNAs regulating gene expression, are frequently aberrantly expressed in human cancers. Next-generation deep sequencing technology enables genome-wide expression profiling of known miRNAs and discovery of novel miRNAs at unprecedented quantitative and qualitative accuracy. Deep sequencing was performed on 11 fresh frozen clear cell renal cell carcinoma (ccRCC) and adjacent non-tumoral renal cortex (NRC) pairs, 11 additional frozen ccRCC tissues, and 2 ccRCC cell lines (n = 35). The 22 ccRCCs patients belonged to 3 prognostic sub-groups, i.e. those without disease recurrence, with recurrence and with metastatic disease at diagnosis. Thirty-two consecutive samples (16 ccRCC/NRC pairs) were used for stem-loop PCR validation. Novel miRNAs were predicted using 2 distinct bioinformatic pipelines. In total, 463 known miRNAs (expression frequency 1–150,000/million) were identified. We found that 100 miRNA were significantly differentially expressed between ccRCC and NRC. Differential expression of 5 miRNAs was confirmed by stem-loop PCR in the 32 ccRCC/NRC samples. With respect to RCC subgroups, 5 miRNAs discriminated between non-recurrent versus recurrent and metastatic disease, whereas 12 uniquely distinguished non-recurrent versus metastatic disease. Blocking overexpressed miR-210 or miR-27a in cell line SKCR-7 by transfecting specific antagomirs did not result in significant changes in proliferation or apoptosis. Twenty-three previously unknown miRNAs were predicted in silico. Quantitative genome-wide miRNA profiling accurately separated ccRCC from (benign) NRC. Individual differentially expressed miRNAs may potentially serve as diagnostic or prognostic markers or future therapeutic targets in ccRCC. The biological relevance of candidate novel miRNAs is unknown at present.

## Introduction

Renal clear cell carcinoma contributes to about 3% of all human cancers [1]. The incidence of the disease has been steadily rising in Europe to over 30,000 new cases per year [2]. Of the various histological subsets, renal clear cell carcinoma (ccRCC) is the most common subtype at diagnosis. One third of patients present with metastases, whereas another third will develop metastases. The majority of patients with distant metastases will succumb to the disease despite introduction of novel effective targeted agents to treat patients with metastatic disease [3,4]. There are no robust diagnostic markers to reliably establish the prognosis at the time of diagnosis in an early stage of the disease.

MiRNAs regulate gene expression post-transcriptionally and have been found to modulate crucial biological processes such as differentiation, proliferation and apoptosis [5]. Dysregulated miRNAs have been reported in many human cancers [6–8]. Next-generation deep sequencing enables miRNA profiling at unprecedented quantitative and qualitative levels. Compared to conventional miRNA array platforms, the major advantages of sequencing technology are massive parallel analysis of genome-widely expressed miRNAs (miRNome), quantification of expression levels of individual miRNAs (absolute abundance), identification of miRNA sequence variations and the discovery of novel miRNAs.

A number of mainly array platform-based studies recently demonstrated that a considerable number of miRNAs are dysregulated in ccRCC [9–17] and a few miRNA have been reported to be functionally involved in ccRCC [18,19]. Although the expression profiling results of the various array studies are not consistent, the data indicate that dysregulated miRNAs may play a pivotal role in the pathogenesis of ccRCC.

At present, there is a need for a quantitative genome-wide miRNA expression profiling using a robust technology to provide better insight of miRNAs dysregulation in RCC. To this end, we performed miRNA deep sequencing in a large number of clear cell RCC tumors and paired NRC to identify dysregulated miRNAs that may serve as reliable diagnostic markers and potential therapeutic targets.

## Results

### Genome-wide Expression of miRNAs

From all sequenced 35 miRNA libraries, we identified a total of 463 miRNA sequences, expressed both in RCC and normal kidney tissues (data available in the Gene Expression Omnibus database http://www.ncbi.nlm.nih.gov/geo: GEO series GSE37616). Of these, 284 miRNA sequences matched to both the 3p-arm and the 5p-arm of 142 miRNA precursors. We also included 94 miRNAs which matched only to a 3p-arm and 85 miRNAs which matched only to a 5p-arm of their miRNAs precursors (Table S1).

The expression level of individual miRNAs within each library varied greatly ranging from 1 to 147,000 sequence read counts per million. In each RCC miRNA library, a small number of miRNAs was abundantly expressed and the same miRNAs were also most abundantly expressed in RCC cell lines and normal kidney miRNA libraries.

The nine most abundant miRNAs (miR-21, miR-145, miR-29a, miR-451, miR-29c, miR-23a, miR-126, miR140 and miR-125a) contributed up to 55%, 48% and 50% of the total miRNA pool in the 22 ccRCC tumors, 2 ccRCC cell lines and 11 normal kidney tissues, respectively.

The twenty most abundantly expressed miRNAs contributed to more than 70% of the total miRNAs of each of these miRNA libraries, whereas the top 50 miRNAs comprised more than 90% of each miRNA library.

### Differentially Expressed miRs in ccRCC Versus Matched NRC

**Figure 1:**
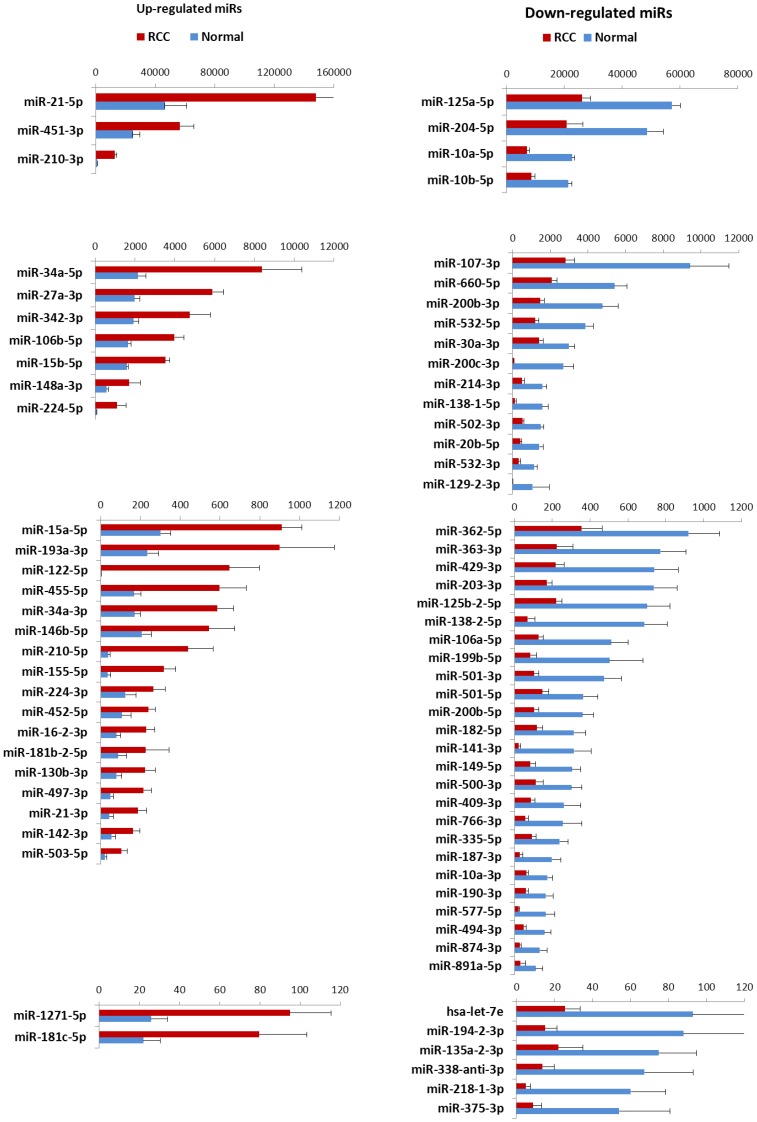
Differentially expressed miRNAs in ccRCC tumors. Differentially expression was analyzed between 11RCC tumors and their matched 11 normal kidney tissues. Significance was determined by adj. P value <0.05 using edgeR package. Differentially expressed miRNAs categorized into four categories based on level of expression. Expression levels are given as Mean ±SD. In the left panels (A-D) overexpressed miRNAs are shown, each panel representing a 10-fold difference in expression level. In the right panels (E-H), downregulated miRNAs are depicted with a 10-fold difference in maximal miRNA expression in normal tissue in each panel.

Significant differential expression of 100 miRNAs was found between 11 ccRCC and NRC after adjustment for multiple testing. After applying additional filtering with a stringent cut-off of 50 read counts/million, 70 miRNAs were robustly differentially expressed. These 70 miRNAs consisted of 29 (42%) miRNAs that were up- and 41 (59%) that were downregulated (adjusted P<0.05). In Figure 1, these 70 miRNAs are depicted according to overexpression in ccRCC and grouped based on expression levels (i.e. upregulated and downregulated miRNAs depicted in the left, respectively right panels).

The most abundantly upregulated miRNAs were miR-21-3p, miR-451-3p and miR-210-3p, of which miR-21 had an expression level in RCC exceeding 140,000 read counts/million. Of the 41 downregulated miRNAs, miR125a-5p, miR-204-5p and miR-10a/b-5p were the most abundantly expressed in the normal kidney cortex tissues.

### Fold-change of Differentially Expressed miRNAs

**Figure 2:**
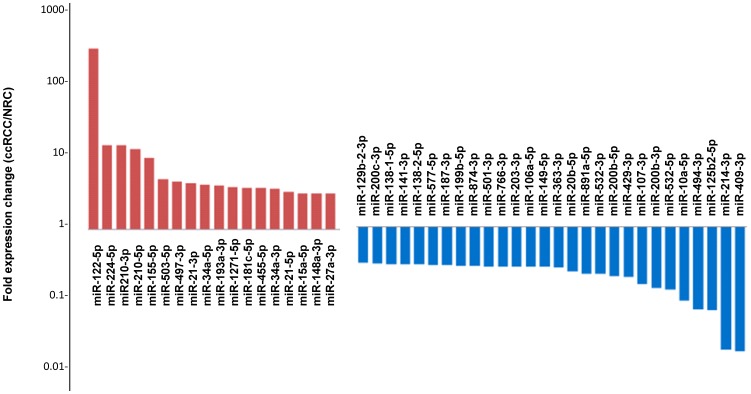
Expression fold-changes of the >3fold differentially expressed miRNAs. The expression levels of the significant miRNA in ccRCC were relative to that of matched normal kidney tissue, as expression fold change. Only miRNAs expression fold-change >3 are presented. The red bars indicate fold-increase of miRNAs in ccRCC compared to normal kidney tissues, the blue bars indicated miRNAs fold increase in miRNA expression in normal kidney versus ccRCC.

With regard to the fold-change in expression levels, 40 differentially expressed miRNAs showed a greater than 3-fold change in expression (Figure 2). Amongst the miRNAs with the greatest fold-change between ccRCC and normal kidney cortex were the upregulated miRNAs miR-122-5p, miR-224-5p, miR-210-5p, the downregulated miR409-3p, and the upregulated miR-21-3p.

### Hierarchical Clustering

**Figure 3:**
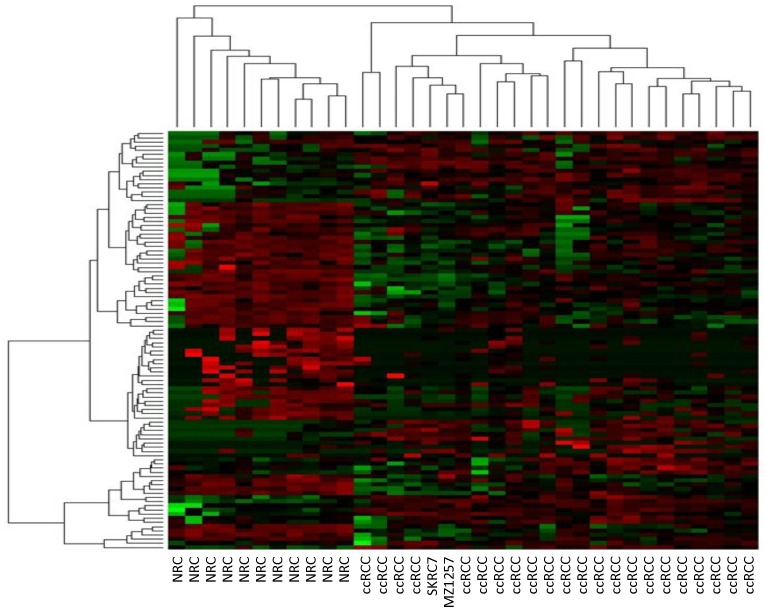
Unsupervised cluster analysis of the 100 differentially expressed miRNA derived from differential expression analysis with 11 ccRCC tumors and matched 11 normal additional 11ccRRC tumors and 2 ccRCC cell lines SKRC andMZ127.

We next performed an hierarchical clustering analysis utilizing the 100 miRNAs differentially expressed between 11 tumor/normal tissue pairs obtained by deep-sequencing to visualize the distinct expression profile between sequenced ccRCC samples and ccRCC cell lines from normal kidney. The expression patterns of the 100 miRNAs clearly separated the 11 normal kidney tissues from the 11 corresponding ccRCC tumors, and the 11 ccRCC tumor samples without corresponding normal tissue, and the cell lines SKRC and MZ1257 clearly clustered with the 11 tumors, and no outliers were obvious (Figure 3).

### PCR Validation of Differential Expression of miRNAs

**Figure 4:**
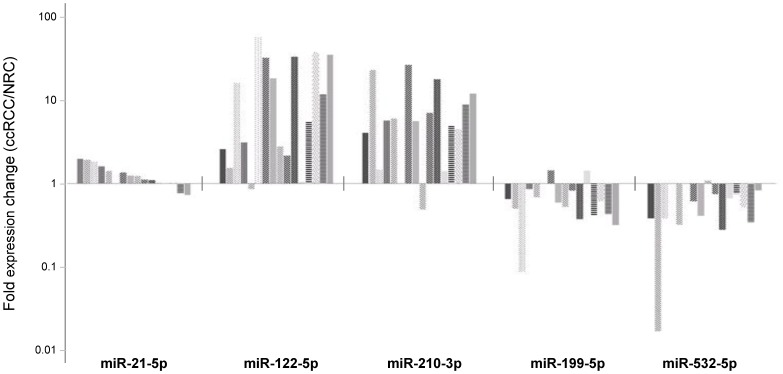
miRNA stem-loop PCR validation. miRNA expression in paired tissue samples of ccRCC and adjacent normal tissue. Small RNA preparations were analyzed for the expression of five selected miRNAs by stem-loop PCR. The expression level of each miRNA was normalized to a small RNA reference U6. The normalized expression values of RCC tumors were relative to the paired normal tissues, and converted expression fold changes. All miRNAs showed statistical significance, determined by pair-wise student t test with P<0.05. Each bar represents one individual RCC tumor.

To validate the deep sequencing results, we performed a stem-loop PCR utilizing 5 miRNAs selected from the 100-miRNA gene expression signature list in 32 additional tissue samples consisting of 16 ccRCC tumors and 16 paired NRC of individual patients. Results of the technical duplicates were very similar. Stem-loop PCR confirmed overexpression of miR-21, miR-122 and miR-210 and downregulation of miR-199 and miR-532 (Figure 4). In accordance with our sequencing results, miR-122-5p and miR-210-3p showed the largest fold-change in expression levels between tumor and normal kidney cortex tissue in agreement with the deep-sequencing findings.

### Expression of miRNA in the ccRCC Prognostic Subgroups

To identify differentially expressed miRNAs among the three ccRCC prognostic sub-groups across 22 patients, we conducted differential expression analysis using the same edgeR package and identified 21 miRNAs distinguishing the 3 sub-groups pairwise. Comparison between the non-recurrent and recurrent sub-group revealed significant expression differences of 9 miRNAs, miR-138-1-5p, miR-181-2-5p, miR-181-5p, miR-182-5p, miR-29b1-3p, miR-29b2-3p, miR193b-3p, miR-15a-3p and miR-1247-5p, five of which also discriminated between non-recurrent and metastatic sub-group.

**Figure 5:**
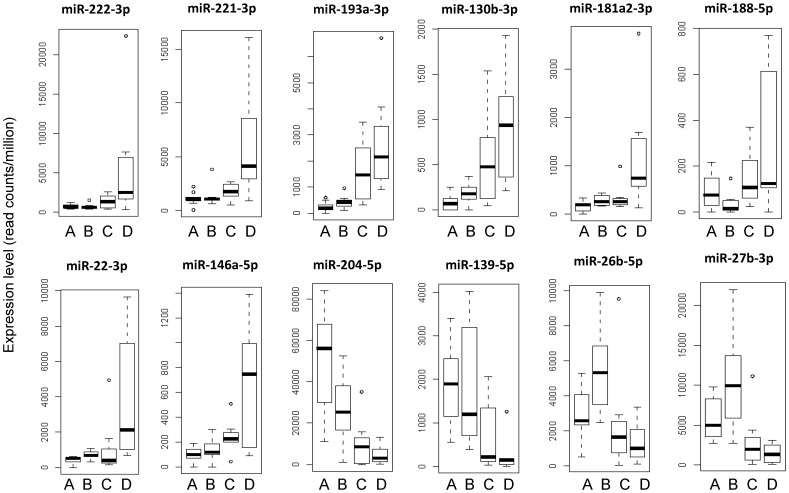
miRNA expression according to deep sequencing analysis. In three RCC patient subgroups with different clinical outcomes (B-D) and adjacent normal kidney tissues (A) derived from 11 of these 25 patients. Statistical significance of miRNA expression in each group was calculated using R package edgeR statistics within 3 RCC subgroups A) no recurrence subgroup (n = 7), C) recurrence subgroup (n = 8) and D) metastatic subgroup (n = 7). The 12 miRNAs depicted were selected based on highly significant difference in expression level in metastatic RCC (D) in comparison to RCC without recurrence (B) (adj. P value <0.05 BH correction). miRNA expression level (read counts/million) is presented as boxplots generated at R.

When comparing the non-recurrent with the metastatic sub-group, a set of 17 miRNAs showed significant expression changes, of which 5 also discriminated between non-recurrent and recurrent sub-group. The remaining 12 miRNAs uniquely discriminated between metastatic and non-recurrent sub-group (Figure 5) and their expression level was overall higher than of the other 9 miRNAs. Of interest, we did the same analysis to identify differentially expressed miRNAs among the three ccRCC prognostic sub-groups based on Leibovich scores but did not find any correlation between miRNA expression and the three Leibovich subgroups in the group of 22 ccRCC patients (data not shown).

### Survival of RCC Patients and miRNA Expression

Next, we performed Kaplan Meier survival analyses to explore a possible association between miRNA expression levels and patient survival. To this end, ccRCC tumors were simply dichotomized using the median miRNA expression level as cut off. Interestingly, dichotomization based on the median of 4 miRNAs, -miR-222-5p, miR-27a-5p, miR-125b-3p and miR-935-3p, albeit their expression levels were rather low, suggested that higher miRNA expression level was associated with poor overall survival (Figure S1).

### Functional Assays of miR-210 and miR-27a

We subsequently performed a limited set of functional analysis of 2 of the PCR-confirmed highly differentially expressed miRNAs, miR-210-3p and miR-27a-3p. Following transfecting the RCC cell line SKRC7 with miR-210 and miR-27a, respectively, we efficiently knocked down both miRNAs as demonstrated by PCR (data not shown). No significant change in percentage of apoptosis or proliferation rate of SKRC7 cells was observed at the various time points measured after knocking down of miR-210 and miR-27a, respectively. No significant enhancement of the percentage of apoptotic cells at 24 and 48 hr as evidenced by Annexin V+/PI+double positivity was observed following addition of anti-miR-210-3p, anti-miR-27a-3p and scrambled control to SKRC7 cells. The percentage of Annexin V+/PI+double positive SKCR7 cells were as follows: 1.62 and 3.9%; 1.28 and 3.62% and 3.58 and 4.50% Annexin V+/PI+ SKCR7 cells, respectively at 24 and 48 hr for anti-miR-210-3p, anti-miR-27a-3p and scrambled control treated SKRC7 cells. No difference was observed in cell viability and proliferation rate between anti-miR-210-3p, anti-miR-27a-3p, and scrambled control-treated SKRC7 cells as measured by WST-1 and counting cell numbers at 24 to 72 hr.

### Differentialy Expressed miRNAs and their Predicted Targets

The miRecord database enabled us to list our differentially expressed miRNAs and the various validated and confirmed targets of such a miRNA for which experimental evidence exist in the litterature. Of the 70 differentially expressed (3p- and 5p-) miRNAs identified by us, targets of 44 precursor miRNAs can be found in miRecord database and these are reported in Table 1. Since often no distinction is made between 3p- and 5p-arms of individual miRNAs we simplified our list of miRNAs to report the targets of the precursor miRNA (Table 1). Using DAVID database enabled us in silico prediction of the combined sets of upregulated or downregulated miRNAs and their potential target networks of genes and signaling pathways (Table 1). We subsequently retrieved the enriched biological pathways predicted by DAVID bio-informatics database and found that the top enriched pathways concern apoptosis and transcription pathways (Table S2).

**Table 1:** Differentially expressed miRNAs in ccRCC of which experimental evidence exist for one or more target genes.*

miRNA	Change	Reported elsewhere	Validated targets (miRecord*)
miR-106b	↑	Ref 13,17	E2F1, VEGFA, CDKN1A, E2F1, ITCH
miR-122	↑	Ref 9,13,16,17	GTF2B, SLC7A1, ALDOA, SLC7A1, AKT, SLC7A1, CCNG1, BCL2L2, in total 50 targets#
miR-130b	↑	Ref 13,17	TAC1
miR-146b	↑	Ref 17	MMP16
miR-148a	↑	-	DNMT3B, NR1I2, Rps6ka5, DNMT1
miR-155	↑	Ref 11,15,16,17	AGTR1, BACH1, LDOC1, MATR3, TM6SF, AGTR1, RHOA,ETS1,MEIS1, in total 24 targets#
miR-15a	↑	Ref 13,17	BCL2, H3F3B, MCL1, VEGFA, DMTF1, RAB21, CADM1, SKAP2, WT1, in total 67 targets#
miR-15b	↑	Ref 17	BCL2, RECK, CCNE1, MKK4
miR-16-2	↑	Ref 10,17	CCND1, TPPP3, BCL2, H3F3B,
miR-181b-2	↑	-	TCL1A, PLAG1, VSNL1, GRIA2, AICDA, ESR1, NLK, CDX2, GATA6
miR-193a	↑	Ref 17	E2F6, PTK2, MCL1
miR-21	↑	Ref 10, 13,17	PTEN, TPM1, E2F1, TGFBR2, CDK6, TIMP3, NFIB, PDCD4, FAS, FAM3, in total 37 targets#
miR-210	↑	Ref 9,11,15,16,17	EFNA3, MNT, CASP8AP2
miR-224	↑	Ref 9,11,15,16,17	API5, KLK10, KLK1, AP2M1
miR-27a	↑	Ref 10	SP3, SP4, PHB, RUNX1, GCA, PEX7,FOXO1, FADD
miR-34a	↑	Ref 15,17	NOTCH1, DLL1, BCL2, E2F3, VEGF, MYCN, SIRT1, CCND1, MYB, JAG1, in total 21 targets#
miR-451	↑	Ref 17	ABCB1, MIF
miR-503	↑	Ref 17	CCND1
miR-106a	↓	Ref 16	VEGFA,RB1, RNUX1, APP, CDKN1A
miR-107	↓	-	PLAG1, CDK6, BACE1, SERBP1, CRKL, RAB1B, AGO3, CDCA4, AGO1, in total of 11 targets#
miR-10a	↓	Ref 13,17	HOXA1, USF2
miR-10b	↓	Ref 17	HOXD10, KLF4
miR-125a	↓	Ref 16,17	LIN28, ERBB2, ERBB3, TP53, HUR, ARID3B
miR-125b-2	↓	Ref 15,16	LIN28, ERBB2, CDK6, H3F3B, CDC25A, BAK1, NTRK3, PERP, GPR160, in total 52 targets#
miR-129-2	↓	Ref 16	NOTCH1, EIF2C3, CAMTA1, BMPR2, SOX4, ZFP91, TP53INP1, FNDC3b, in total 12 targets#
miR-135a-2	↓	Ref 15	APC, FLAP, JAK2
miR-138-1	↓	Ref 9,13,16	RHOC, KRT, ROCK2
miR-141	↓	Ref 9,13,15,16	SERBP1, SFPQ, HMGB1, CLOCK, TGFB2, ELMO2, WDR37, SIP1, KLHL20, in total 13 targets
miR-182	↓	Ref 13,17	RARG, ADCY6, MITF, IGF-IR, WAVE
miR-199b	↓	Ref 9,16	LAMC2, SET
miR-200b	↓	Ref 10,11,13,15,16,17	ZEB1, ZEB2, RERE, ELMO2, ERRFI1, KLHL20, FOG2, WAVE3, BAP1, in total 13 targets#
miR-200c	↓	Ref 9, 11,13,15,16,17	ZEB1, ERRFL1
miR-203	↓	Ref 13	SOCS3, TP63, ABL1
miR-204	↓	Ref 15,17	ARPC1B, SPDEF, CTSC, MMP3, MMP9, BMP1, CDH11, ITGB4, SHC1, in total 19 targets#
miR-20b	↓	-	VEGFA, ESR1
mir-214	↓	Ref 13,16	PTEN
miR-218-1	↓	Ref 11	LAMB3, COL1A1, ECOP, SP1
miR-30a	↓	Ref 15,16,17	NOTCH1, TNRC6A, BECN1P1, KRT7
miR-335	↓	Ref 16	SOX4, PTPRN2, MERTK
miR-375	↓	Ref 13,16	YAP1, YWHAZ
miR-429	↓	Ref 11,16	RERE, ELMO2, ERRF11, ERBB2IP, KLHL20, WRD37, PTPRD, BAP1, FOG2
mir-494	↓	-	PTEN
miR-502	↓	Ref 16	SET8
miR-532	↓	Ref 13,16,17	RUNX3

Of the 70 differentially expressed miRNAs with >50 read counts/million found by us only the 44 miRNA of which a validated target is present in miRecord are reported.

* Above experimentally proven targets are listed in miRecord target database version 3. miRecord does not allow appropriate distinction between 3p- ad 5p- forms of each miRNA due to differences in techniques applied in the various reports in the literature.

# If more than 9 targets per miRNA are reported, only nine are enlisted in the Table.

In addition, we applied another database miRror, which contains in silico predicted, non-validated, as well as validated predicted targets, to identify specific targets of the 70 differentially expressed miRNAs, either the 3p- or 5p-, and retrieved the enriched pathways by DAVID. These targets and predicted pathway results are different (data not shown) and we rely on the miRecord database validated targets (Table 1) and derived predicted pathways using DAVID (Table S2). The limitation of using different methods, in particular using databases containing non-validated targets, is that different pathway predictions may result.

### Novel miRNA Prediction

Using two independent bioinformatics pipelines, we predicted 121 candidate miRNAs from all the sequenced samples which meet the criteria of stem-loop secondary structure of miRNA precursor and have not been reported before to be expressed in humans or other species. Of the 121 novel miRNA, 23 miRNAs showed expression levels >50 read counts/million (Table S3). None of the predicted novel miRNAs showed a significant differential expression between ccRCC and NRC and these candidate miRNAs have not been validated yet.

## Discussion

Using state-of-the-art sequencing technology, we have quantified 463 genome-widely expressed miRNAs in ccRCC, normal kidney and ccRCC cell lines. Overall, the miRNome of ccRCC tumors resembled that of the ccRCC cell lines. Quantitative differential expression analysis allowed the identification of a 100-miRNA signature distinguishing ccRCC from normal kidney. We identified 21 miRNAs discriminating tumors of favorable prognosis patients (non-recurrent) from those with a less favorable prognosis of which patients with metastatic disease at time of diagnosis had the worst prognosis and all died within 1.5 years from RCC; four other miRNAs seemed associated with disease-specific death. Quantitative deep-sequencing technology allowed the discovery of novel miRNAs which have not been reported before in any species and were shown to be present in normal kidney tissue and tumors.

Of the most abundantly expressed miRNAs, miR-21, miR-451, miR-125a and miR-204 together contributed to 27% and 18% of total miRNAs in ccRCC and normal kidney tissue. MiR-21, the most abundantly expressed miRNA accounting for 14% of the total miRNA in ccRCCs and 5% in normal kidney, has been found to be overexpressed in many human cancer and to act as an oncogene by targeting tumor suppressor PTEN in various cancers [20]. Of interest, the highly overexpressed MiR-451 and miRNA-27a have been reported to regulate multi-drug resistance (MDR) in carcinoma cell lines [21], and since RCC often express the MDR phenotype these two miRNAs might indeed be important in contributing to the RCC phenotype.

One hundred of 463 (21%) genome-widely expressed miRNAs showed significant expression changes in RCC. Recently, dysregulation of miRNAs in ccRCC has been reported [9–17]. Of these studies, 7 studies made use of array-based platforms, one of a miRNA PCR-primer array platform [11] whereas only Weng et al [16] applied deep sequencing technology to assess whether expression profiles in 3 ccRCC differed between stored frozen versus fixed samples. The latter investigators found that formalin-fixed paraffin-embedded tissue could be used to profile small RNAs. Mostly, small numbers of ccRCC tumors were used for expression profiling. Some studies used normal kidney as control tissue, other studies compared miRNA expression profiles between ccRCC and different kidney tumor histologies.

Our data confirm the ccRCC miRNAs findings reported in the literature [9–17] (Table 1) but there are also discrepancies with regard to reported dysregulated miRNAs in other studies and the dysregulated miRNAs found by us utilizing deep sequencing technology. For instance, Nakada et al [9] have reported 43 miRNAs differentially expressed in ccRCC and 15 miRNAs were also found by us. Chow et al [13] reported 80 differentially expressed miRNAs of which only 15 overlapped with our 100 miRNAs. Despite the large extent of overlapping results, the discrepancies between (combined) results from earlier array studies and our current deep sequencing results may arise from differences in sample preparation, different technologies and the quantitative accuracy of the applied deep sequencing technology. However, the direction of the differential expression of miRNAs found by us and others did not differ except for the fact that one study did not report the direction for all of the dysregulated miRNAs. Moreover, in our study a highly stringent selection criterion of ≥95% tumor cell content was used for miRNA library construction which may further enhanced the sensitivity of miRNA detection.

A multitude of studies have investigated the mRNA targets of one single miRNAs and the comprehensive, publicly available miRecord database enabled us to identify the various currently validated targets of the list of differentially expressed miRNAs as identified by us. Using DAVID database enabled us in silico prediction of the combined sets of upregulated or downregulated miRNAs and their potential target networks of genes and signaling pathways. Target and pathway prediction suggested involvement of the deregulated miRNAs in a variety of biological processes involved in malignant behavior of cells and in the transformation of normal cells to malignant cells. To understand functional implications of the target genes, enriched biological pathways predicted according to DAVID bio-informatics database were identified. Interestingly, the top enriched pathways are apoptosis and transcriptions. The deregulated miRNAs and their top enriched pathways were in particular involved in apoptosis and transcription suggesting that these deregulated miRNAs may indeed play roles in a variety of biological processes involved in malignant behavior of cells and in the transformation of normal cells to malignant cells. Based on this integrative approach, our data provide an important platform for future investigations aiming at characterizing the role of specific miRNAs in ccRCC pathogenesis.

We tested whether upregulated miRNAs could be efficiently knocked down by using antagomirs and this was affirmed. The second question was, whether knocking down of upregulated miRNAs would affect proliferation and/or apoptosis in one RCC cell line used. Both miR-210 and miR-27a were linked to predicted target genes which are of interest with regard to cell proliferation and apoptosis (see Table 1).

Our exploratory experiments showed that proliferation and apoptosis of SKRC7 cells were not affected by knocking down miR-210 or miR-27a following transfection with specific antagomirs. Data in other tumor types suggested that miR-210 is indeed involved in proliferation. Interestingly, upregulation of miR-210 was also reported in other malignancies, including breast and head and neck cancer, and found to correlate with prognosis [22,23], although in some studies downregulation of miR-210 was found in cancer, e.g. breast cancer in comparison with benign breast epithelium and esophageal cancer [24]. Expression of miR-210 might merely reflect the hypoxia status known to be typically present in ccRCC and serve as a surrogate marker for tumor hypoxia because miR-210 is the most robustly induced miRNA under hypoxia [18,22,23]. This might explain the lack of effect on proliferation or apoptosis in our cell line miR-210 knock down experiments, but differences in miR-210 target expression of FGFRL1 might be another explanation.

Two other studies suggested that miR-27a may be involved in the development of tumor drug resistance [25] and we were interested in miR-27a because of its involvement in MDR/P-glycoprotein expression in cancer cells, a typical characteristic of many RCCs.

The lack of effect on proliferation and/or apoptosis after knocking down miR-210 and miR-27a in one ccRCC cell line may indicate that the effect of these miRNAs on the endpoints chosen do not apply to ccRCC and/or to this particular cell line only, or that the conditions to affect proliferation and/or apoptosis require a more intricate interplay of more factors. Discrepancies between individual cancer cell lines and tumors may be determined by cell-specific differences in expression levels of miRNA target genes or other cellular c.q. exogenous factors.

Of the dysregulated miRNAs miR-122 showed the largest fold-change in expression level in RCCs. Although expression level of miR-122 is much lower than that of miR-21, the clear cut expression change in RCC suggests that it may serve as a novel biomarker to distinguish tumor from normal kidney tissue. Notably, 5 downregulated miRNAs miR-532 and miR-362, miR-500, miR-501, miR-502 are clustered and together encoded in one intron of a renal specific gene voltage-gated chloride ion channel CLCN5, and have been reported to be downregulated in ccRCC [26].

Differentially expressed miRNAs as we found in our limited subgroup of metastatic RCC not only serve as potential prognostic markers, but also even as possible therapeutic targets. Of the 12 miRNAs which discriminated between non-recurrent and metastatic prognostic sub-groups, miR-221/222 has been reported in many other types of human cancers including prostate carcinoma [27]. MiR-130b has been suggested to regulate expression of the tumor suppressor gene RUNX3 [28] whereasmiR-146a has been shown to play an important role in oncogenic transformation of immune cells in mice model [29].

The downregulated miR-204, which has been found to be also dysregulated head and neck cancer [30], displayed very high expression in NRCs with expression level of on average 5.8% (range 1.1 to 8.2%), of the total miRNAs, but gradually decreased following the sub-groups order of no recurrence, recurrence and metastatic sub-groups. Of interest, miR-204, located on chromosome 9 in intron 6 of the potential Ca2+ channel TRPM [31] may proof to be a robust classifier if validated in an independent set of ccRCC/NRC samples.

A key advantage of deep sequencing is that it is a powerful tool allowing the discovery of novel miRNAs that cannot be detected using array-based technology. The use of our deep sequencing data and two additional pipelines allowed discovery of 21 novel miRNAs which generated perfect secondary structures indicating that deep sequencing is indeed a powerful tool to identify novel miRNAs of as yet unknown function. Future studies are warranted to proof that they are bonafide miRNAs.

### Conclusion

Characterization of the miRNome of clear cell renal cell carcinomas by deep sequencing enabled to precisely quantify expression levels of miRNA and identify dysregulated miRNAs in RCC that may serve as novel diagnostic marker. Several of the differentially expressed miRNAs potentially target networks of genes and signaling pathways that may be involved in the malignant transformation of normal kidney cells and pathophysiology of ccRCC. Our data provide an important platform for future investigations aimed at characterizing the role of specific miRNAs in ccRCC pathogenesis.

## Materials and Methods

### Patients and Tumors

RCC specimens were used from a total of 38 patients who underwent an open (partial) nephrectomy for ccRCC between1997–2003 at the University Hospital of Leuven, Belgium.

Immediately after surgery, tumor and matched non-malignant kidney tissue were (separately) stored frozen at −80°C according to a standard procedure.

Tumor staging was performed using the 2010 TNM staging system. None of the patients received systemic anti-cancer treatment prior to surgery. Criteria applied to select tissues for deep sequencing and validation by PCR were a sufficiently large sample size based on pre-specified expected variations to allow for firm conclusions within the limitation of costs and financial resources, the time period of diagnosis and minimum of 5 year follow up period of the patients, availability of ccRCC and paired adjacent non-tumoral renal parenchyma called “nontumoral renal cortex” (NRC), and a high yield of high quality RNA in all tissues subjected to deep sequencing and to PCR validation. Central review of tissue blocks were performed by one experienced uro-pathologist and only blocks with >95% viable tumor (ccRCC) or non-tumoral cortex (NRC) were included in this study.

The criteria to select normal (non-tumoral) kidney tissues were that the normal kidney cortex tissue was from the same individual, from the same kidney, from the same surgical specimen, and collected under the same Standard Operation Procedure (SOP) conditions: if possible the sampling of normal cortex was done leaving at least 1 cm of macroscopically healthy cortex adjacent to the tumor untouched. ccRCC are macroscopically characterized by a sharp demarcation (fibrous pseudocapsule) between tumor and surrounding non-tumoral tissue. This is also reflected microscopically, as RCC are sharply demarcated from the surrounding tissue. The non-tumoral tissue was therefore obtained in macroscopically clear-cut benign tissue and immediately fresh-frozen. An Hematoxylin-Eosin stained slide was then made of this frozen tissue block. To be selected as adequate for RNA isolation, the benign nature of the tissue had to be confirmed microscopically. Significant necrosis or extensive inflammation were also excluded out in this way. This was done by one experienced uropathologist (EL).

Deep sequencing and PCR validation of tumors and NRC were performed by members of the team, blinded for the clinical data, without prior knowledge of the TNM stage and clinical outcome of the patients.

In total, 22 RCC tumors and 11 matched non-malignant kidney tissues and 2 RCC cell lines SKRC7 and MZ1257 were used for miRNA deep sequencing, whereas 32 tissues consisting of 16 paired ccRCC and kidney cortex (NRC) were used for PCR validation. The 22 RCC tumors selected for deep-sequencing come from 22 patients with a median age of 61 years old, range, 43–79 years old, of who 7 never had a recurrence of the disease, 8 patients who had a recurrence of the disease and 7 patients presented with distant metastases at time of diagnosis who underwent a cytoreductive (radical) nephrectomy (Table S1).

These 22 deep sequenced RCCs were classified based on known clinicopathological characteristics such as tumor size, Fuhrman grade, nodal involvement, and the widely used prognostic Leibovich score for all patients even though the nomogram is normally only used for non-metastasized tumors [32]. Six tumors fell into the Leibovich favorable (i.e., 1–2 unfavorable risk factors), 6 intermediate (i.e. 3–5 unfavorable risk factors) and 10 poor category (i.e., 6 or more unfavorable risk factors) but the Leibovich categories did not overlap with the three subgroups classification made based on absence or presence of tumor recurrence and distant metastases (see below). These 22 ccRCC patients had namely a priori been classified by the clinicians, all blinded for the deep sequencing and PCR outcome, into patients who never experienced disease recurrence (non-recurrent sub-group, n = 7, of whom 1 died of the disease), patients who progressed after initial nephrectomy into a stage with clinical recurrence of the disease (recurrent disease sub-group, n = 8, of whom 2 died of RCC), and patients who had metastatic disease with distant metastasis at time of diagnosis and cytoreductive surgery (metastatic sub-group, n = 7) and who all died within 1.5 years from RCC.

### Ethics Statement

The regulations of the Ethics Committee of the Leiden University Medical Center (LUMC) and of the Faculty of Medicine, University Hospital of Leuven, permit the research to be conducted without obtaining written informed consent from the patients. The tissue material has been anonymized and the research with the anonymous biological material and its results are not retraceable to the individuals concerned.

### Deep Sequencing

RNA was isolated from approximately twenty 50 µm cryosections prepared at −20°C. Total RNA was isolated using a Trizol reagent (Invitrogen, Carlsbad, CA). RNA with good quality e.g. A260/A280>1.8 was used for further experiments. Of each sample, 2 ug total RNA was used for small RNA separation <200 bp (mirVana miRNA Isolation Kit, Applied Biosystems, Nieuwerkerk a/d IJssel). Next, miRNA libraries were constructed primarily based on the SREK protocol (SOLiD Small expression Kit, Applied Biosystems, Nieuwerkerk a/d IJssel) with modifications as reported elsewhere [33]. Of each small RNA library, the fraction with an estimated size corresponding to RNA of 15 nt to 30 nt was excised from the gel, representing the miRNA library. The miRNA libraries were examined with DNA chip (BioAnalyzer 2100, Agilent Technologies, Palo Alto, CA, USA). Subsequently, the miRNA libraries were subjected to deep sequencing using a Genome Analyzer II (Illumina Inc, San Diego, CA, USA) according to manufacture protocol [33].

### Data Analysis

After the completion of sequencing, the raw sequence data were processed through the standard Illumina pipelines for base-calling and fastq file generation. The sequence reads were mapped to the human reference genome GRC37 using a bioinfomatic E-miR pipeline [33]. According to human genome annotation, mapped reads were classified into non-coding RNA species e.g. miRNA, mtRNA, and rRNAs (Table S4). We defined reads as miRNA if they mapped to the miRNA precursor. The small RNA library contained in majority of miRNA, with a proportion ranging from 64–74% in matched normal kidney tissue, from 68–81% in RCC and of 77 and 74%, respectively in RCC cell lines, indicating consistency of miRNA sample preparation (Figure S2).

miRNA expression level was quantified by relative miRNA read counts to the total read counts of all miRNAs per library (expressed as counts/million). miRNA differential expression analysis was performed with Bioconductor edgeR package 1.6, in which we used an overdispersed Poisson model [34] with a common dispersion parameter, combined with the exact test. For testing differential expression of ccRCC with matched NRC we used a paired analysis. We also performed differential expression analysis of deep sequenced miRNAs in the three clinical subgroups as well as in the three groups based on the Leibovich score. The three categories of clinical subgroups and Leibovich score were tested in a pairwise fashion. The significant miRNAs were determined by an adjusted P value <0.05 based on the Benjamini and Hochberg multiple testing correction [35]. Fold changes were calculated using ratios of the arithmetic mean of the normalized miRNA counts within each group.

### Clustering Analysis

The significant miRNAs with log2 transformed expression levels were subjected to hierarchical cluster analysis in R with Euclidean distance and complete linkage.

### Stem-loop PCR

We performed stem-loop PCR to validate the results of the deep sequencing analysis for five selected differentially expressed miRNAs. Total RNA was extracted from RCC tumors and matched controls from additional 16 patients. Of each sample, 150 ng total RNA was used for miRNA cDNA synthesis (Megaplex Pool A 2.0, Applied Biosystems, Nieuwerkerk a/d IJssel). Stem-loop PCR (Taqman MicroRNA Assays, Applied Biosystems Nieuwerkerk a/d IJssel) was performed on a LightCycler 480 (Roche diagnostics, Almere, Netherlands) with a thermal program of 95 C/20 s, 60 C/40 s for 40 cycles and of each sample a technical duplicate was performed. The miRNA expression level was normalized to reference RNA RNU44. Fold expression change was determined based on the delta Ct method [36].

In the PCR validation set, there was a similar distribution with regard to clinical subgroups as in the deep-sequencing set of 22 tumors.

### Kaplan-Meier Survival Analysis

For each miRNA sequenced, the 22 RCC patients were divided into 2 groups according to expression level ≥ median (high level) or < median (low level). The relationship between cancer specific death and miRNAs expression level was analyzed by Kaplan Meier analysis. A Cox regression model was applied using square root transformed counts. P<0.05 was considered significant, and no correction for multiple testing was applied.

### Transfection

Anti-miRNA inhibitor (100 nM) of either miR-210-3p or miR-27a-3p, respectively, or scrambled Negative Control (100nM, Applied Biosystems) with fluorescently labeled oligonucleotides (100 nM, BLOCK-iT, Invitrogen) was transfected into a renal cell line SKRC7 by Lipofectamine 2000 (Invitrogen). All experiments were performed in duplicate. In all experiments, the transfection efficiency was 90% based on percentage of BLOCK-it fluorescence positive CKRC7 cells. Tumor cell apoptosis (Annexin V staining) was evaluated 24 hr and 48 hr after transfection as previously described [37]. Proliferation rate was assessed at 24, 48 and 72 hr post transfection with either antagomir or scrambled control by assessing cell viability (WST-1 reagent) and counting the number of viable cells using trypan blue dye.

### Novel miRNA Prediction

For novel miRNA identification the HHMMiR [38] and microPred [38] tools were used. Sequences from all tumor and normal samples that were not annotated to any of the non-coding Ensembl transcripts were extracted from the data files. Sequence regions with length between 16 and 24 nucleotides and with expression of at least 5 sequence reads were selected for further analysis. For each of the remaining candidate regions the 40 nt upstream and downstream flanking sequences were retrieved via the Ensembl perl API. The novel microRNAs predicted by both HHMMiR and microPred tools were presented.

### miRNA Target Prediction and Enriched Pathway Analysis

miRNA target genes were retrieved from the comprehensive, publicly available miRecord database (www.mirecord.org). For each of the differentially expressed miRNA, only experimentally proven target genes were selected. Based on the target genes, the enriched pathways were predicted at bioinformatics DAVID database (http://david.abcc.ncifcrf.gov).

In addition, miRNA target genes were retrieved from the publicly available miRror database (http://www.proto.cs.huji.ac.il/mirror/), target genes selected for each of the 70 individual, differentially expressed miRNAs and by linking to the DAVID database, enriched pathways were predicted.

## Supporting Information

Figure S1 **miRNA expression and ccRCC patients survival.** Kaplan-Meier survival plots were generated using Cox regression model. P<0.05 was considered significant, and no correction for multiple testing was applied (PDF file).
(TIFF)

Figure S2 **The sequenced RNA reads were mapped to Human Genome and subsequently classified into variant RNA species.** The total read counts of each RNA species was expressed as percentage (Mean±SD) of the total read counts per sequenced library (PDF file).
(TIFF)

Table S1 **Clinical information and normalized reads of sequenced miRNAs in each sequenced sample.** The expression level is presented as read count/million (DOC file).
(XLSX)

Table S2 **Enriched biological pathways predicted using DAVID database.** The experimentally proven targets were retrieved from miRecord database and subsequently used for enriched pathways prediction analysis (XLS file).
(XLSX)

Table S3 **Novel miRNA candidates were predicted using two independent HMMiR and microPred.** These are novel miRNAs not previously reported in any species. The novel miRNA candidate with expression levels exceeding 50 read counts/million in all sequenced samples are presented (XLS file).
(XLS)

Table S4 **Quality and composition of sequenced small RNA libraries (XLS file).**
(XLSX)
