# Supplementary figures and images for: Genome-Wide MicroRNA Expression Analysis of Clear Cell Renal Cell Carcinoma by Next Generation Deep Sequencing
Source: PLoS One. 2012 Jun 20;7(6):e38298. doi: 10.1371/journal.pone.0038298 (PMC3380046; doi:10.1371/journal.pone.0038298)

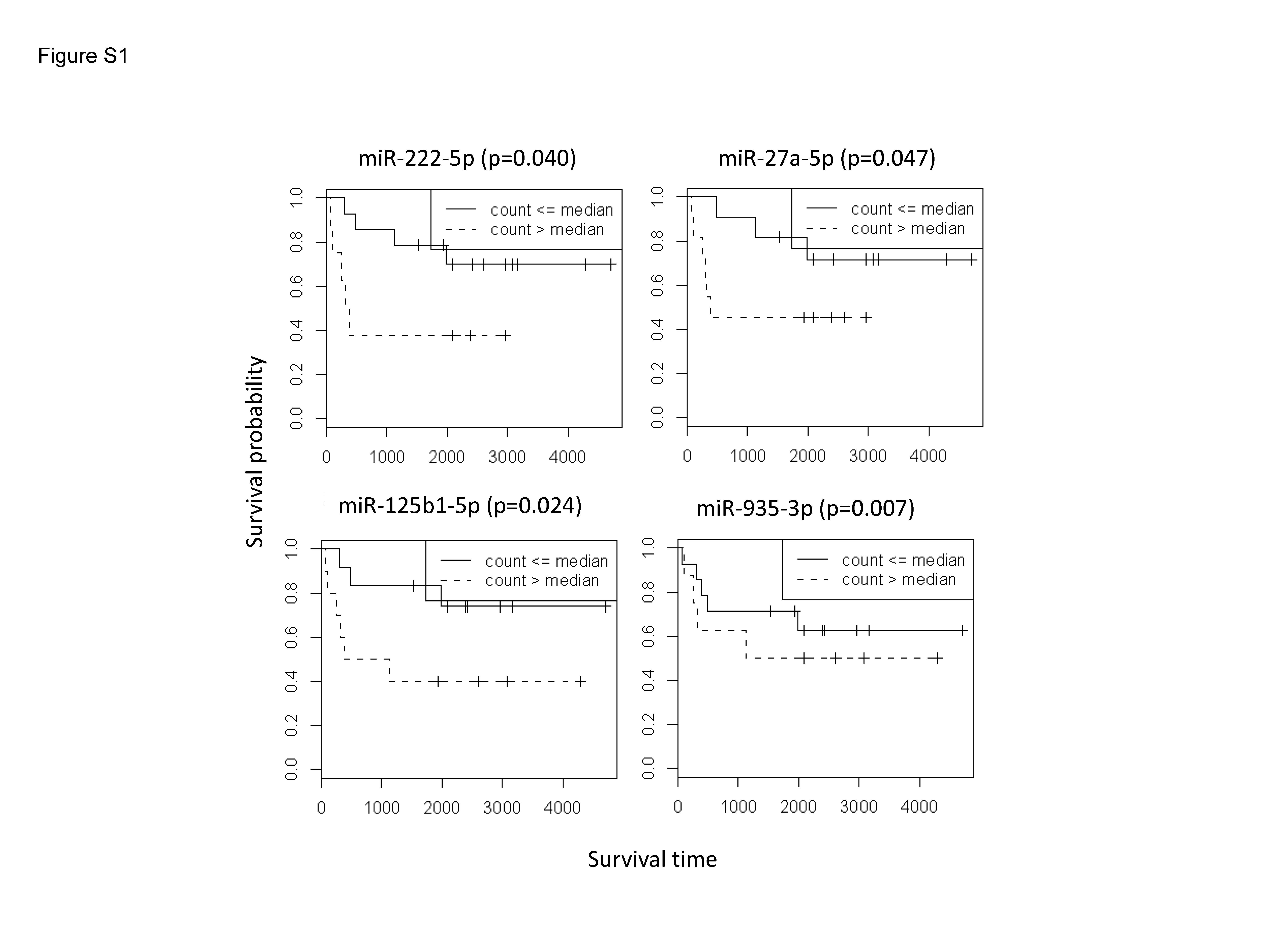

Supplement: Figure S1 — miRNA expression and ccRCC patients survival. Kaplan-Meier survival plots were generated using Cox regression model. P<0.05 was considered significant, and no correction for multiple testing was applied (PDF file). (TIFF) [file pone.0038298.s001.tiff]

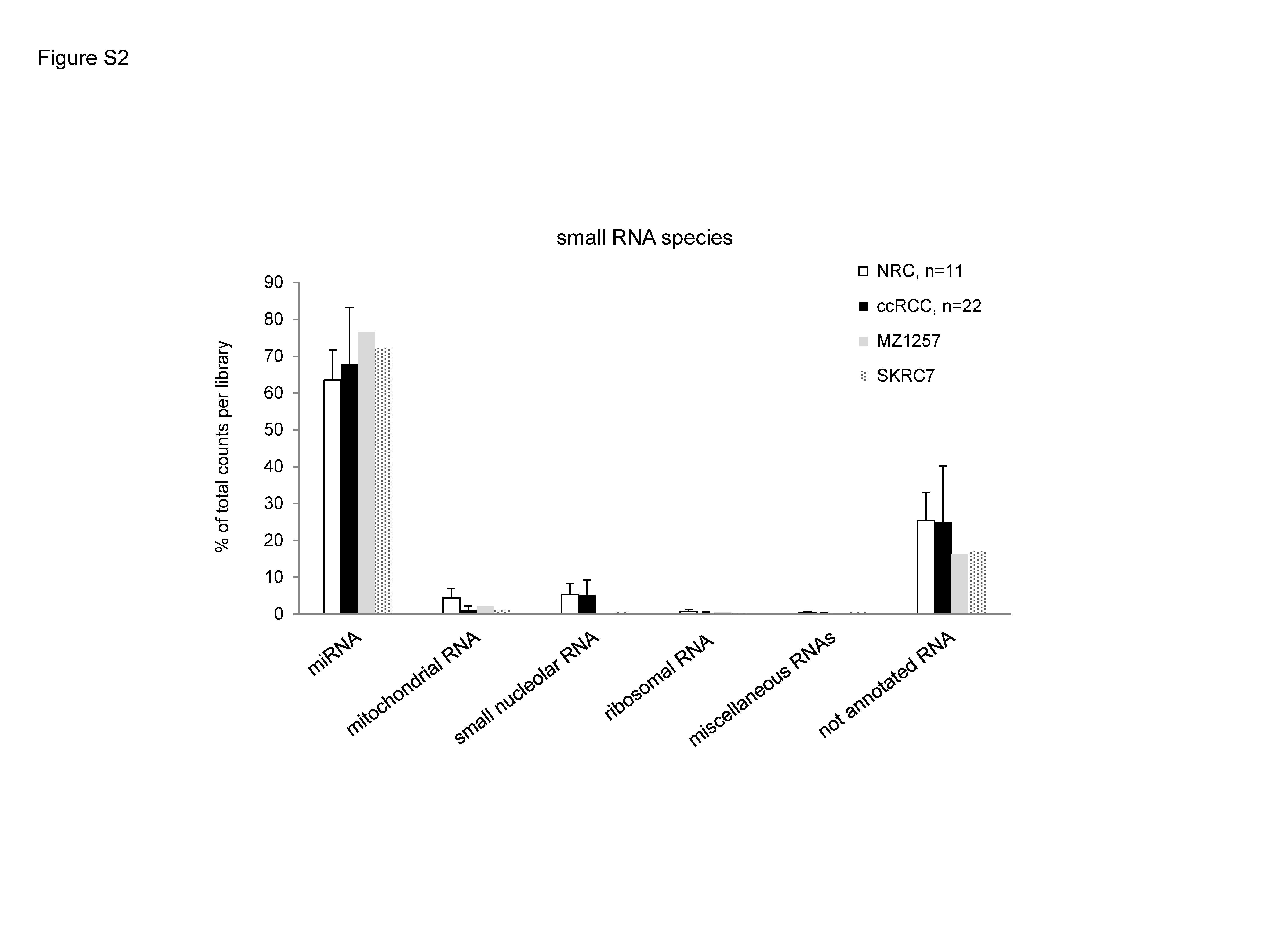

Supplement: Figure S2 — The sequenced RNA reads were mapped to Human Genome and subsequently classified into variant RNA species. The total read counts of each RNA species was expressed as percentage (Mean±SD) of the total read counts per sequenced library (PDF file). (TIFF) [file pone.0038298.s002.tiff]
